# miRNA326-5p Targets DKC1 Gene to Regulate Apoptosis-Related Proteins and Intervene in the Development of Neuroblastoma

**DOI:** 10.1155/2023/6761894

**Published:** 2023-07-01

**Authors:** Xiao-Hui Wang, Shu-Feng Zhang, Hai-Ying Wu, Jian Gao, Lin Wang, Xu-Hui Wang, Tian-Hui Gao

**Affiliations:** ^1^Department of Pediatric Surgery, Henan Provincial People's Hospital, Zhengzhou 450000, China; ^2^Department of Obstetrics, Henan Provincial People's Hospital, Zhengzhou 450000, China; ^3^Department of Medical Oncology, Henan Provincial People's Hospital, Zhengzhou 450000, China

## Abstract

**Objective:**

To study the effect of congenital dyskeratosis 1 (DKC1) on neuroblastoma and its regulation mechanism.

**Methods:**

The expression of DKC1 in neuroblastoma was analyzed by TCGA database and molecular assay. NB cells were transfected with siDKC1 to observe the effects of DKC1 on proliferation, cloning, metastasis, and invasion, and apoptosis and apoptosis-related proteins. The tumor-bearing mouse model was constructed, shDKC1 was transfected to observe the tumor growth and tumor tissue changes, and the expression of DKC1 and Ki-67 was detected. Screening and identification of miRNA326-5p targeting DKC1. NB cells were treated with miRNA326-5p mimic or inhibitors to detect the expression of DKC1. NB cells were transfected with miRNA326-5p and DKC1 mimics to detect cell proliferation, apoptosis, and apoptotic protein expression.

**Results:**

DKC1 was highly expressed in NB cells and tissues. The activity, proliferation, invasion, and migration of NB cells were significantly decreased by DKC1 gene knockout, while apoptosis was significantly increased. The expression level of B-cell lymphoma-2 in shDKC1 group was significantly lower than that of the control group, while the expression level of BAK, BAX, and caspase-3 was significantly higher than that of the control group. The results of experiments on tumor-bearing mice were consistent with the above results. The results of miRNA assay showed that miRNA326-5p could bind DKC1 mRNA to inhibit the protein expression, thereby inhibiting the proliferation of NB cells, promoting their apoptosis, and regulating the expression of apoptotic proteins.

**Conclusion:**

miRNA326-5p targeting DKC1 mRNA regulates apoptosis-related proteins to inhibit neuroblastoma proliferation and promote the apoptotic process.

## 1. Introduction

Neuroblastoma (NB) is one of the most common malignant solid tumors in childhood, accounting for about 7% of childhood malignant tumors and 15% of childhood malignant tumor deaths. In recent years, the incidence of NB has increased rapidly, and the 2-year survival rate of children after diagnosis is only 38% [1, 2]. NB is mainly manifested as mass and appears in the tissues of sympathetic nervous system [3]. It is highly malignant and prone to bone marrow metastasis and distant metastasis at early stage, which is the main cause of death in NB children. Currently, the main clinical treatment methods of NB include surgery, radiotherapy, chemotherapy, and immunotherapy [1, 4–6], but the therapeutic effect is still unsatisfactory and the prognosis is poor. Therefore, it is particularly important to elucidate the mechanism of NB growth, proliferation, and metastasis, and to provide evidence for the prevention or inhibition of tumor by corresponding intervention measures.

With the rapid development of biotechnology, a large number of omics data, such as genes [7] and proteins [8], have emerged, which contain complex disease information. By integrating multiple omics data through bioinformatics, potential key genes, and regulatory pathways such as tumors can be mined, which is conducive to in-depth understanding of the underlying mechanism of the disease and facilitate subsequent clinical application [9, 10]. At present, The Cancer Genome Atlas (TCGA) is the most complete and largest tumor gene information database at home and abroad. This database not only contains relatively comprehensive cancer types, but also includes comprehensive multiomics data information. In this study, the gene congenital dyskeratosis 1 (DKC1) related to the survival analysis of neuroblastoma was screened from TCGA tumor database [11] by data mining. Preliminary experimental studies showed that the gene was highly expressed in neuroblastoma tissues and cell lines. Studies have shown that DKC1 is dysregulated in a variety of tumors, including glioma, breast cancer, and nonsmall cell lung cancer, but it is less well-studied in neuroblastoma. Therefore, we explored the mechanism of DKC1 in neuroblastoma through in vitro and in vivo experiments, in order to provide a new target for the treatment of neuroblastoma.

## 2. Materials and Methods

### 2.1. TCGA Database

Neuroblastoma data were obtained from TCGA tumor database using CGDSR package in R language. OS_MONTHS and OS_STATUS were screened from the data for KM survival function, including progression-free survival and overall survival. The time distribution of events is as follows: “Deceased” and “Living.”

### 2.2. Clinical Samples

Tumor tissue and paracancer tissue samples of 12 patients with neuroblastoma diagnosed by Henan Provincial People's Hospital were collected. All included patients had complete clinical data. Fresh tissues were collected and frozen with liquid nitrogen for reserve.

This study was approved by the Drug Clinical Trial Ethics Committee of Henan People's Hospital, complying with relevant guidelines and regulations. It mainly involves the collection of pathological tissues from patients with neuroblastoma and all participants provided written informed consent after complete de-ion of the study. All methods were carried out in accordance with relevant guidelines and regulations.

### 2.3. Cell Culture

Neuroblastoma cell lines CHP-126, CHP-134, SH-SY5Y, SK-N-SH, LAN-1, and IMR-32 were procured from ATCC, USA. NB cells were cultured in F12/DMEM medium (Solarbio, Beijing, China) with 10% FBS, at 37°C and 5% CO_2_ incubator. DKC1 si-RNA, miR-326-5p inhibitor, miR-326-5p mimic, DKC1 mimic, and corresponding control vector (Jiuruiwu, Henan, Zhengzhou, China) were transfected into neuroblastoma cells, respectively, with Lipofectamine 3000 reagent (Semmerfeld, USA), and the operation was carried out strictly in accordance with kit instructions.

### 2.4. Real-Time PCR Assay

RNA was extracted from tissues (cells) by trizol-chloroform-isoamyl alcohol method (Thermo, USA), cDNA was synthesized by reverse transcription kit (Servicebio, Wuhan, China), and the relative expression of DKC1 was detected by real-time quantitative PCR. Primer sequences were designed on Primer 5.0 software and synthesized by Shenggong Bioengineering (Shanghai, China). Primer sequences are shown in Table 1. PCR reaction system consists: 2x qPCR Mix 12.5 *μ*l, 2.5 *μ*M Primer 0.5 *μ*l, antisense transcripts 2.0 *μ*l, ddH2O 4.0 *μ*l.

### 2.5. Western Blot Assay

Western blot was used to detect protein expression. Protein lysate (Jingcai, Shanghai, China) was used to lysate cells and extract total protein from cells. Twenty micrograms protein was added to sample loading buffer and boiled for 20 min. Centrifugation was performed at 4°C and 12,000 × *g* for 10 min. Precipitation was discarded and analyzed by SDS–PAGE (Jingcai, Shanghai, China). After electrophoresis, membrane transfer was performed at 200 mA for 90 min. Mass fraction 5% skim milk—tris buffer solution-tween (TBST) sealed at room temperature for 2 hr. Primary antibody : TBST membrane was washed three times, 8 min each time, and primary antibody was incubated overnight at 4°C (antibody dilution ratio : DKC1 : 1:1,000 dilution; GAPDH diluted at 1 : 10,000) (wanleibio, Shenyang, China). Second antibody: remove the membrane of the incubated primary antibody, restore to room temperature, wash the membrane three times with TBST, 8 min each, and incubate the second antibody for 2 hr at room temperature (sheep antirabbit IgG combined with horseradish peroxidase 1 : 1,000) (Wanleibio, Shenyang, China). Development was performed with ECL luminescent solution. The film is scanned and the gray value of the target strip is analyzed using a Gel image processing system (Gel-Pro-Analyzer software).

### 2.6. CCK-8 Assay

After treatment of cells, the culture medium was removed and replaced with 100 *μ*l fresh medium in each well. Next, 10 *μ*l CCK-8 was added and cells were incubated at 37°C for 1 hr. The OD value at 450 nm was measured on a microplate analyzer, and data analysis was performed.

### 2.7. Colony Formation Assay

Cells in each group at logarithmic growth stage were digested with 0.25% trypsin and beaten into single cells, respectively. After dilution, cells were placed in a cell incubator at 37°C, 5% CO_2_, and saturated humidity for 2 weeks. Discard the supernatant, soak the cells in phosphate-buffered saline (PBS) twice, and fix the cells with 5 ml 4% paraformaldehyde for 15 min. Finally, GIMSA was added and dyed with dyeing solution for 10–30 min. Count under microscope (low magnification). Clone formation rate = (number of clones/number of inoculated cells) × 100%.

### 2.8. Transwell Assay

Transwell assay was performed using modifified Boyden chambers (Transwell, USA). Trypsin was added in the petri dish for digestion, and cells were adjusted to the appropriate density for observation and counting. In 24-well plates, 400 *μ*l complete medium was added to the lower chamber, and 200 *μ*l serum-free cell suspension was added to the upper chamber, and then cultured in an incubator. After 24 hr culture, paraformaldehyde was fixed for 25 min, saline was cleaned for three times, and crystal violet was added for 40 min staining. The multifunction camera takes pictures and records the measured values.

### 2.9. Wound Healing Assay

Add an appropriate amount of cells to the 24-well plate. After 24 hr, compare the ruler with the head of the gun, and try to hang the horizontal scratches on the back. The cells were washed three times with PBS and added serum-free medium. Put it into a 37°C, 5% CO_2_ incubator, and culture. Samples were taken at 0, 6, 12, and 24 hr.

### 2.10. Experiment on Tumor-Bearing Mice

Twelve 5-week-old nude mice was subcutaneously injected with neuroblastoma CHP-134 cells. A group of six mice were transfected with shDKC1 lentivirus plasmid or control plasmid. After inoculation, tumor formation of nude mice was observed every 7 days and measured with calipers to record growth data. On Day 28, nude mice were sacrificed, tumor tissues were collected and weighed, and placed in formalin for use.

### 2.11. HE Detection

Formalin was fixed in the tissues for 48 hr for dewatering and wax immersion. Embedding machine for embedding. Cut the tissue into 3–5 *μ*m thick slices. Baking machine for baking: 70°C, 30 min; water bath: 49°C, 20 s; dewaxing and rehydration: Toluene1 10 min, Toluene2 10 min, anhydrous ethanol 1 5 min, anhydrous ethanol 2 5 min, 90%/80%/70% anhydrous ethanol (5 min each), distilled water 3 min. Hematoxylin nuclear staining: hematoxylin staining for 4 min, and running water washing for 3 min; differentiation (75% anhydrous ethyl alcohol + 1% 1 ml hydrochloric acid) for 5 s, and washing with running water for 3 min; eosin was dyed for 20 s and washed with running water for 2 min; bake for 5 min at 70°C; seal slices. Microscopic examination, image collection, and analysis were performed.

### 2.12. IHC Detection

Paraffin section routine dewaxing. Antigen repair : 1x EDTA, microwave oven medium heat 8 min, ceasefire 7 min, medium to low heat 7 min, cool to room temperature, wash three times in PBS, 5 min each. Sealing: after the tissue was circled with SP pen, BSA was added (diluted to 3% by PBS) and sealed for 50 min. Incubating primary antibody: add primary antibody, 4°C overnight; Incubating secondary antibody: wash with PBS three times, 5 min each, add secondary antibody DAKO, and incubate at room temperature for 50 min. DAB color rendering: PBS 10 min (shaking table), 1x DAB, pay attention to avoid light, microscope observation. Hematoxylin nuclear staining : running water washing for 1 min, hematoxylin staining for 2 min, and running water washing for 1 min; differentiation + cyanosis (75% anhydrous ethyl alcohol 100 ml + 1% hydrochloric acid 1 ml) for 2-3 s, and washing with running water for 1 min; bake for 5 min at 70°C; seal slices. Microscopic examination, image collection, and analysis were performed.

### 2.13. Flow Cytometry

Cell apoptosis was detected by staining with Annexin V-FITC/PI. Cells were collected by centrifugation and the supernatant was discarded; cells were washed twice with PBS and 500 *μ*l binding buffer was added. Next, 5 *μ*l Annexin V-FITC and 10 *μ*l propidium iodide were added and cells were incubated at room temperature in the dark for 5–15 min. Analysis by flow cytometry was performed.

### 2.14. Dual Luciferase Reporter Assay

Te target gene of miR-326-5p was identifed using a dual luciferase reporter assay. First, the pmiR-RB-Report vector with wild-type 3′-UTRs of DKC1 (wt) or mutant DKC1 3′-UTRs (mut) were obtained from RiboBio (China). Ten, wt, or mut reporter constructs and miR-326-5p mimics or inhibitors were co-transfected into NPC cells with Lipofectamine 3000 (invitrogen). After 48 hr of incubation, passive lysis bufer was use to lyse the cells, which were further assayed using the Dual Luciferase Reporter Assay kit (Promega, USA) according to the manufacturer's protocol. Renilla luciferase assay was used to normalize the luciferase activity of each lysate.

### 2.15. Data Analysis

SPSS 23.0 and GraphPad Prism5.0 were used to analyze and plot the experimental data. *T* test was used to compare the measurement data between groups. *P* < 0.05 indicated statistical significance.

## 3. Results

### 3.1. Expression of DKC1 in Neuroblastoma Tumor Tissue

By analyzing the differential genes of neuroblastoma in TCGA database and screening the genes related to the survival of patients with neuroblastoma, we found that DKC1 gene was associated with the overall survival of patients (*P* < 0.01) and progression-free survival time (*P* < 0.01) were significantly correlated (Figure S1).

**Figure 1:**
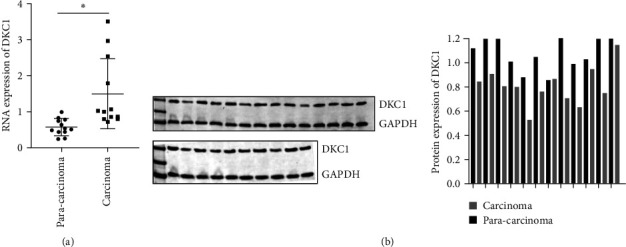
Expression verification of DKC1 in clinical tissue samples. (a) mRNA expression level verification. (b) Verification of protein expression level.

The mRNA and protein expression levels of DKC1 in tumor tissues and adjacent tissues of 12 clinical neuroblastoma cases were detected. The results showed that the mRNA and protein levels of DKC1 in tumor tissues were significantly higher than those in adjacent tissues, as shown in Figure 1. The high expression of DKC1 in tumors suggests that it may be closely related to neuroblastoma.

### 3.2. DKC1 Is Involved in Neuroblastoma Regulation

DKC1 expression, including mRNA level and protein level, was detected in human neuroblastoma cell line CHP-126 and five neuroblastoma cell lines CHP-134, SH-SY5Y, SK-N-SH, LAN-1, and IMR-32. The results showed that the mRNA and protein levels of DKC1 in five neuroblastoma cells were significantly higher than those in CHP-126 cells, especially in CHP-134 and LAN-1 cells (Figure S2). We selected CHP-134 and LAN-1 cell lines with high expression as the research objects for subsequent in vitro experiments.

**Figure 2:**
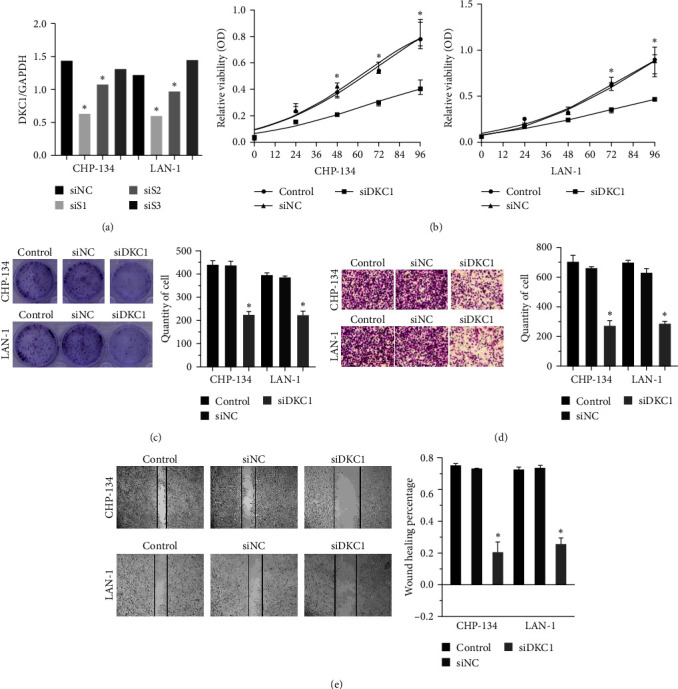
Downregulation of DKC1 expression inhibits tumor development in CHP-134 and LAN-1 cells: (a) Selection of DKC1 siRNA, after transfection with DKC1 siRNA; (b) CCK-8 detected the activity of CHP-134 and LAN-1 cells; (c) cloning experiment; (d) the invasion of CHP-134 and LAN-1 cells was detected by transwell assay; (e) the cell migration of CHP-134 and LAN-1 was detected by scratch assay.

We selectively knocked out DKC1 in CHP-134 and LAN-1 cells, respectively (Figure 2(a)). Results of CCK-8 assay, colony formation assay, transwell assay, and wound healing assay showed that DKC1 knockdown resulted in decreased cell activity, proliferation, invasion, and migration in CHP-134 and LAN-1 cells (Figure 2(b)–2(e)).

**Figure 3:**
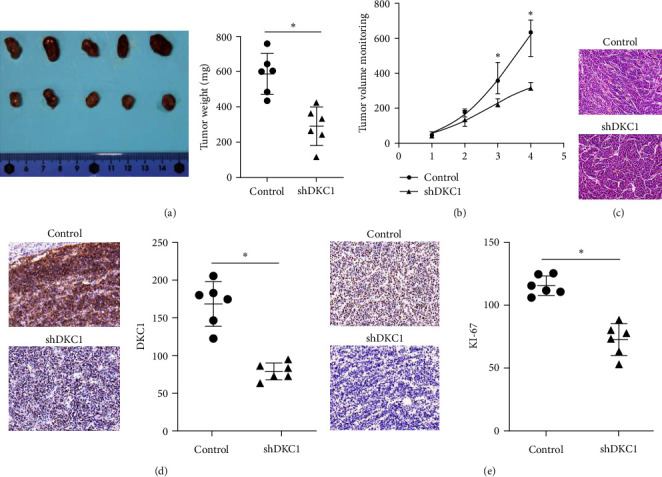
Downregulation of DKC1 expression inhibits tumor development in mice: (a) Measurement of tumor growth state; (b) tumor growth curve analysis; (c) HE staining analysis of tumor tissue; (d) IHC detection of DKC1 in tumor tissue; (e) IHC detection of KI-67 in tumor tissue.

In addition, we studied the development of NB tumor in vivo, and the results showed that when DKC1 was knocked out, the tumor volume was significantly smaller than that of the control group (Figure 3(a)), and the tumor growth curve was significantly lower than that of the control group (Figure 3(b)). The transplanted tumor tissues were stained with hematoxylin and eosin, and observed under light microscope (Figure 3(c)). The transplanted tumor tissues in each group were wrapped with fibrous connective tissue, and the interstitial edema was obviously knocked out. Compared with the NC group, the tumor necrosis area of keratinocyte carcinoma increased nuclear fission. Immunohistochemistry of DKC1 in transplanted tumor tissue showed that the expression of DKC1 in shDKC1 group was significantly lower than that in NC group (Figure 3(d)). Proliferative nuclear protein Ki-67 is one of the most widely used proliferative cell markers, which can reflect the proliferative activity of tumor cells and be used to judge the degree of malignancy of tumor cells [12, 13]. In this study, immunohistochemical detection of Ki-67 in the transplanted tumor tissues of the two groups showed that the expression of Ki-67 in siDKC1 group was significantly lower than that in NC group (Figure 3(c) and 3(e)), and downregulation of DKC1 expression could inhibit the expression of Ki-67 and tumor proliferation.

### 3.3. DKC1 Interferes with Neuroblastoma Apoptosis by Regulating Apoptosis-Related Proteins

**Figure 4:**
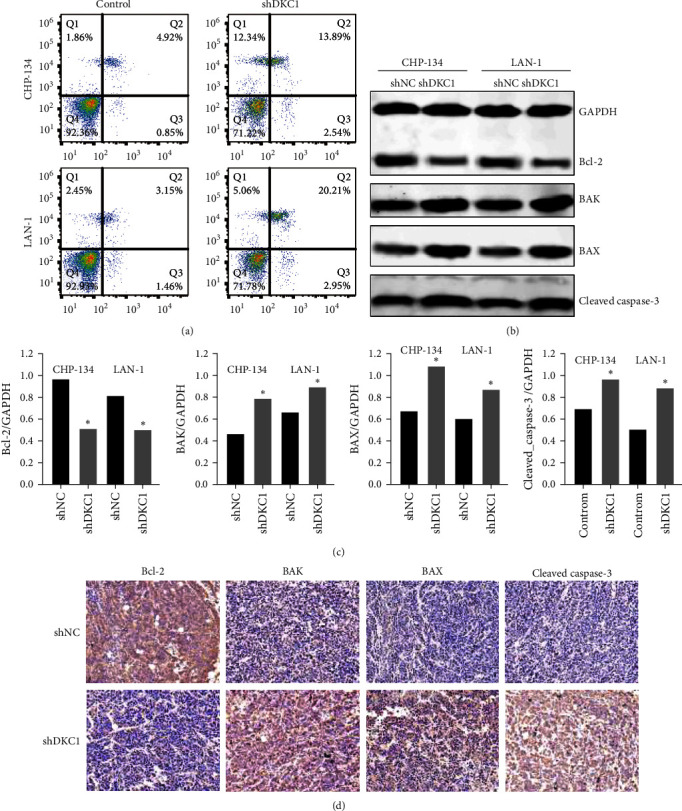
Downregulation of DKC1 expression promotes apoptosis of neuroblastoma cells. After DKC1 was knocked out, (a) flow cytometry was used to detect cell apoptosis, and (b and c) Western blot was used to detect apoptosis-related proteins, and (d) IHC was used to detect apoptosis related protein levels.

Flow cytometry analyses of Annexin-V/PI double staining were performed in order to investigate the effect of DKC1 on cell apoptosis of CHP-134 and LAN-1 cells as shown in Figure 4(a). Compared with the control group, the apoptosis of CHP-134 and LAN-1 cells was significantly increased in the shDKC1 group. B-cell lymphoma-2 (Bcl-2), BAK, BAX, and caspase-3 are well-known apoptosis-related proteins, among which Bcl-2 is an antiapoptotic factor [14], while BAK, BAX, and caspase-3 are proapoptotic factors [15, 16]. Western blot and IHC were used to detect the expression of the above apoptosis factors. As shown in Figure 4(b)–4(d), the expression level of Bcl-2 in the shDKC1 group was significantly lower than that in the control group, while the expression level of BAK, BAX, and caspase-3 was significantly higher than that in the control group.

### 3.4. miRNA326-5p Targets DCK1 to Regulate the Proliferation and Apoptosis of Neuroblastoma Cells

**Figure 5:**
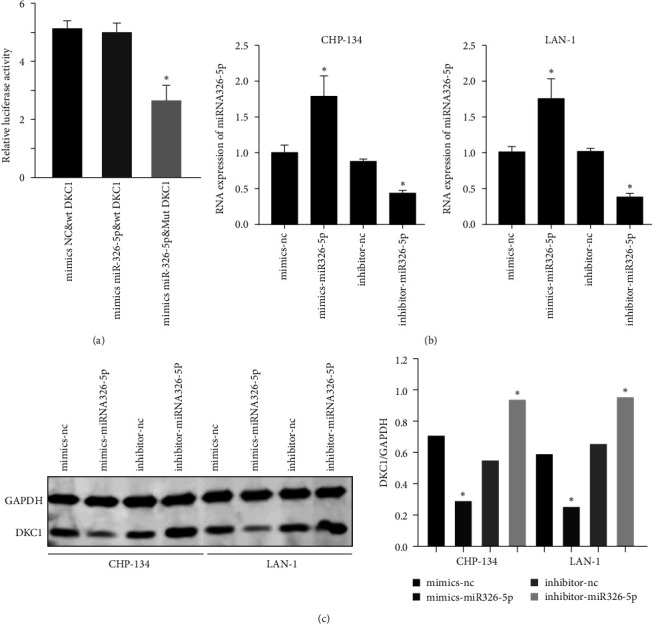
miRNA326-5p regulates DKC1 expression: (a) Double luciferase activity to detect the binding of miRNA326-5p and DKC1; (b) RT-PCR to detect the effect of miRNA326-5p mimic or inhibitor; (c) Western blot detection of DKC1 expression in NB cells treated with miRNA326-5p mimic or inhibitor.

The potential miRNA binding sites of DKC1 mRNA were analyzed using the following databases: TargetScan PitTar and Dia-Microt, and the results showed that miRNA326-5p matched exactly with DKC1 mRNA. Dual luciferase reporter assay showed miRNA326-5p binds DKC1 mRNA (Figure 5(a)). After treat NB cells with miRNA326-5p mimic or inhibitor, DKC1 expression level was also significantly decreased or increased (Figure 5(b) and 5(c)).

**Figure 6:**
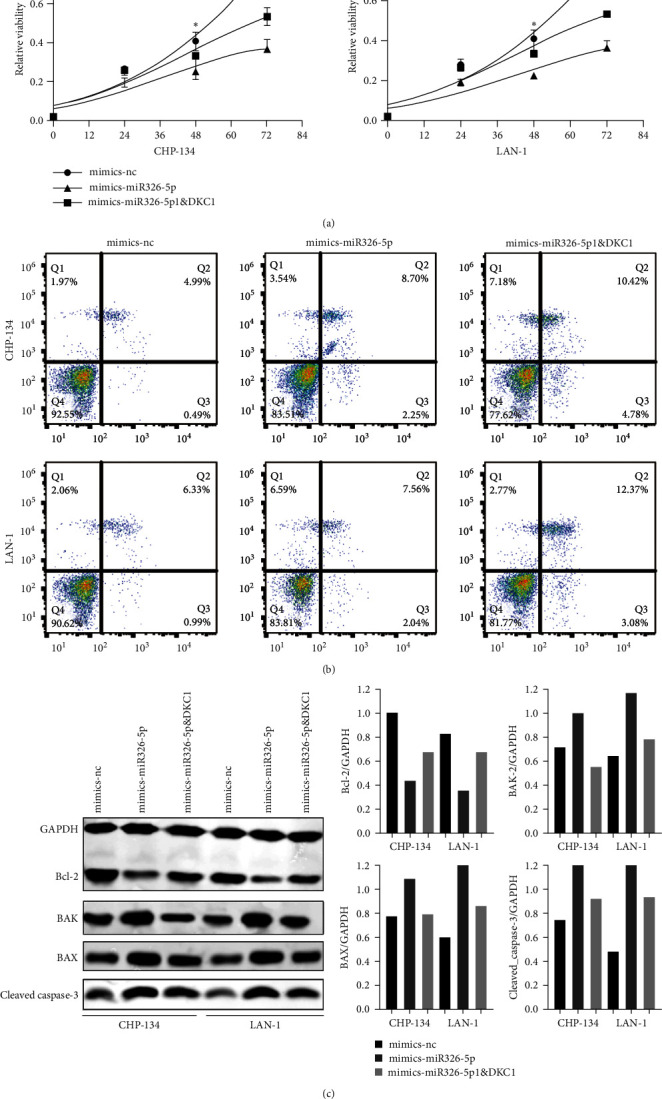
miRNA326-5p interferes with proliferation and apoptosis of NB cells by regulating DKC1 expression. After miRNA326-5p and DKC1 were overexpressed in NB cells: (a) CCK-8 detected the proliferation of cells in each group; (b) cell apoptosis in each group was detected by flow cytometry; (c) the expression of apoptosis-related proteins in each group was detected by Western blot. Mimic-nc was a blank control group, mimic-miRNA326-5p was miRNA326-5p overexpression group, mimic-miRNA326-5p&DKC1 was miRNA326-5p and DKC1 simultaneously overexpression group.

CCK-8 results were shown in Figure 6(a). After 48 hr culture, the proliferation of NB cells in miRNA326-5p mimic group was significantly lower than that in mimic-nc group, while the proliferation of NB cells in miRNA326-5p&DKC1 mimic group was lower than that in mimic-nc group but higher than that in miRNA326-5p mimic group. The results of flow cytometry analysis were shown in Figure 6(b). The apoptosis rate of miRNA326-5p mimic group was significantly higher than that of mimic-nc group, while miRNA326-5p&DKC1 mimic group was significantly higher than that of miRNA326-5p mimic group. Western blot was used to detect the expression of apoptosis-related proteins in the three groups. The results showed that the expression of Bcl-2 in miRNA326-5p mimic group was significantly lower than that in mimic-nc group, while the expression of Bcl-2 in miRNA326-5p&DKC1 mimic group was significantly higher than that in miRNA326-5p mimic group. However, the expression levels of BAK, BAX, and cleaved caspase-3 in miRNA326-5p mimic were significantly higher than those in mimic-nc group, and they in miRNA326-5p&DKC1 mimic group were significantly lower than those in mimic miRNA326-5p (Figure 6(c)).

## 4. Discussion

Pediatric malignant neuroblastoma belongs to the embryonic tumor group and originates from the progenitor cells of the sympathetic adrenal system [17]. Treatment options for children with high-risk and recurrent diseases are still very limited [18]. In recent years, analysis of neuroblastoma gene profiles has revealed increasing molecular diversity, suggesting that molecule-targeted therapy may be a promising treatment option [19].

The DKC1 gene is located on Xq28 on the X chromosome. Dyskerin encoded by DKC1 gene is associated with the formation of certain small RNAs and telomerase activity [20]. Genetic mutations in DKC1 inactivate keratin and lead to congenital dyskeratosis, which is characterized by skin defects, hematopoietic dysfunction and increased susceptibility to cancer [21]. Current studies have shown that DKC1 is dysregulated in a variety of tumors. Studies have shown that the expression of DKC1 is significantly increased in the pathological tissue of glioma, and the expression of DKC1 is related to the tumor stage of glioma. Upregulation of DKC1 in gliomas is common and necessary for extensive tumor growth [22]. DKC1 also has similar manifestations in breast cancer [23], nonsmall cell lung cancer [24], and colorectal cancer [25]. The expression and function of DKC1 in neuroblastoma are rarely reported. Only one report suggested that high DKC1 expression was strongly associated with adverse evnts and overall survival (*P* < 0.0001), the attenuation of keratin inhibits the proliferation and growth of tumor cells [26]. In vitro experiments showed that the cell activity, proliferation, invasion, and migration of NB cells were significantly decreased after DKC1 deletion. In vivo experiments showed that tumor growth of DKC1 knockout mice was slower and the volume was significantly smaller than that of the control group. Proliferative nuclear protein KI-67 is one of the most widely used proliferative cell markers, which can reflect the proliferative activity of tumor cells and can be used to judge the degree of malignancy of tumor cells [12, 13]. In this study, downregulation of DKC1 expression could inhibit the expression of KI-67 and tumor proliferation. Therefore, DKC1 may be involved in the molecular mechanism of neuroblastoma cell proliferation, apoptosis, invasion, and migration.

Flow cytometry was used to detect the apoptosis of neuroblastoma cells. After DKC1 was knocked out, the apoptosis of neuroblastoma cells was significantly increased. Proteins of the Bcl-2 family control the internal apoptosis pathway [27]. Bcl-2 is one of the most important apoptosis inhibiting proteins in the Bcl-2 protein family. The Bcl-2 associated X protein (BAX) has been identified as a homologous binding partner of Bcl-2. Compared with Bcl-2, Bax promotes apoptosis under stress stimulation. The Bcl-2 antagonist killer 1 (BAK) is also a natural counterpart of survival promoting Bcl-2 protein [28, 29]. At steady state, prosurvival Bcl-2 members (e.g., MCL-1 and Bcl-XL) restrain the activity of prodeath BAK and BAX. In response to developmental cues and stress signals, prosurvival activity is overwhelmed, permitting BAK and BAX activation. BAK and BAX oligomerize in the mitochondrial outer membrane, causing its permeabilization and the release of apoptogenic factors, of which the best characterized is cytochromec [30, 31]. Cytoplasmic cytochrome c forms part of the apoptosome complex, which successively activates caspase-9 and the apoptotic effector caspases, caspase-3, and caspase-7. Together, these proteases efficiently cleave a multitude of substrates within the cell to accelerate its demise [32]. However, the regulatory effect of the above-mentioned apoptotic proteins is not constant. Recently, the newly emerged antitumor drug Casiopeinas induces the uncoupling of respiratory chain, generates reactive oxygen species (ROS), Bax enters mitochondria, Ca2+, and Bcl-2 exit from mitochondria, leading to cell apoptosis. And, recent studies have shown that only BAX is necessary for Casiopeína IIIia (CasIIIia)-induced apoptosis [33]. Western blot and IHC assay were used to detect the expression of the above apoptosis factors. The results showed that after DKC1 was knocked out in NB cells, the expression level of Bcl-2 was also significantly inhibited, while the expression levels of BAK, BAX, and caspase-3 were significantly increased. We speculated that DKC1 could regulate Bcl-2 family proteins in neuroblastoma and control the internal apoptosis pathway of tumor cells.

MicroRNAs (miRNAs) are a class of noncoding RNAs with 19–25 nucleotide length. More and more evidences show that miRNAs play a core role in the carcinogenesis of NB [34]. A study identified differentially expressed genes (DEG and DEM) in NB high-risk patients and nonhigh-risk patients through comprehensive bioinformatics analysis, and found that ADRB2 may affect the survival status of high-risk patients due to the regulation of miR-30a-5p [35]. Malregulation of miRNA expression has also been observed in NB cases, and abnormal expression of specific miRNA has been found to be related to the metastasis and invasion of NB cells [36]. In addition, some miRNAs have been shown to play a role in influencing tumor progression by regulating tumor-associated protein MYCN. MiR-15a-5p, miR-15B-5p, and miR-16-5p can intervene in neuroblastoma by inhibiting the expression of MYCN [37]. Another study found that exosome derived from natural killer (NK) cells carrying the tumor suppressor miR-186 was cytotoxic to MYCN-amplified neuroblastoma cell lines. Moreover, MiR-186 was downregulated in high-risk neuroblastoma patients, its low expression is a poor prognostic factor directly related to NK activation markers. The expression of MYCN, AURKA, TGFBR1, and TGFBR2 is directly inhibited by miR-186, which can inhibit the tumorigenetic potential of neuroblastoma [38]. We used bioinformatics tools to analyze the potential miRNAs of DKC1 and found that miRNA326-5p matched DKC1 mRNA perfectly. After overexpression of mirNA326-5P or deletion of miRNA326-5p, the expression level of DKC1 was also significantly decreased or increased, indicating that miRNA326-5p can target the expression of DKC1. CCK-8 results showed that the proliferation of NB cells in the miRNA326-5p overexpression group was significantly lower than that in the control group, while the proliferation rate of cells in the miRNA326-5p and DKC1 overexpression group was significantly higher than that in the miRNA326-5p overexpression group, again confirming the targeted regulation of miRNA326-5p and DKC1. The detection of apoptosis-related proteins was similar to the above results, that is, miRNA326-5p overexpression could inhibit the expression of antiapoptotic protein Bcl-2 and promote the expression of proapoptotic protein by inhibiting the expression of DKC1. However, the overexpression of DKC1 showed the opposite result.

In conclusion, the high expression of DKC1 in neuroblastoma can regulate the proliferation, apoptosis, invasion, and metastasis of neuroblastoma. miRNA326-5p may intervene the proliferation of NB cells by regulating the expression of DKC1, and regulate the apoptosis-related proteins Bcl-2, BAK, BAX, and cleaved caspase interfered with apoptosis of NB cells.

## Figures and Tables

**Table 1:** Primer sequence table.

Name	Primer sequence
DKC1 forward primer	5′-GCTAAGTTGGACACGTCTCAG-3′
DKC1 reverse primer	5′-TGCAAGAGGTGTATAGTGTGTTG-3′
GAPDH forward primer	5′-TGACTTCAACAGCGACACCCA-3′
GAPDH reverse primer	5′-CACCCTGTTGCTGTAGCCAAA-3′

## Data Availability

The datasets generated during and/or analyzed during the current study are available from the corresponding author on reasonable request.

## Supplementary Materials

Supplementary Materials Figure S1: survival analysis of DKC1 gene in TCGA database. Figure S2: high expression of DKC1 in human neuroblastoma cell lines.
