# Supplementary material for: miRNA326-5p Targets DKC1 Gene to Regulate Apoptosis-Related Proteins and Intervene in the Development of Neuroblastoma
Source: Anal Cell Pathol (Amst). 2023 Jul 1;2023:6761894. doi: 10.1155/2023/6761894 (PMC10329557; doi:10.1155/2023/6761894)
Supplement: Supplementary Materials — Figure S1: survival analysis of DKC1 gene in TCGA database. Figure S2: high expression of DKC1 in human neuroblastoma cell lines. [file 6761894.f1.docx]

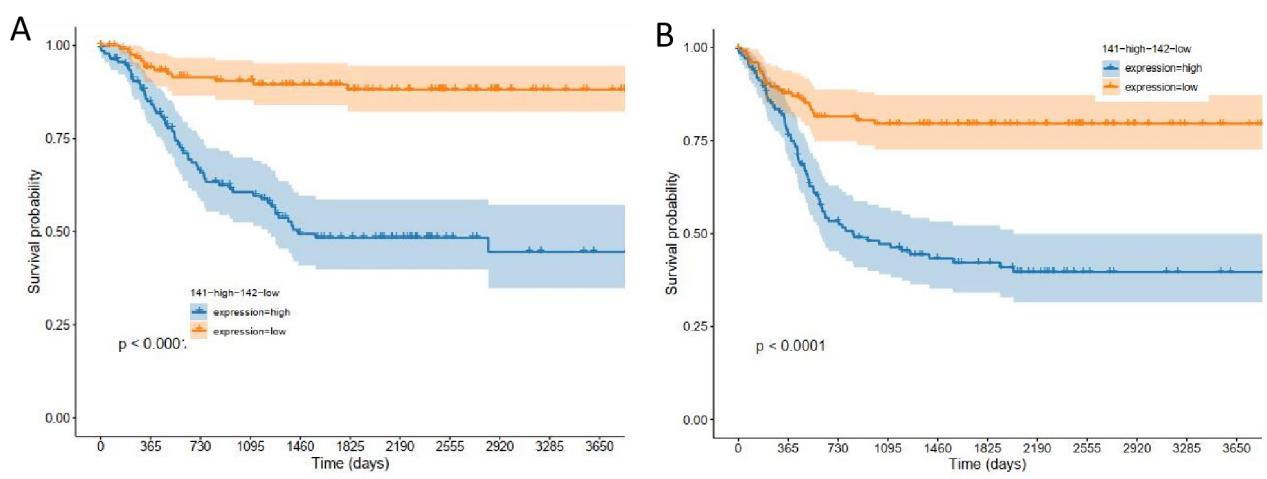


Figure S1 Survival analysis of DKC1 gene in TCGA database. A) Overall survival analysis; B) Progression-free survival time analysis


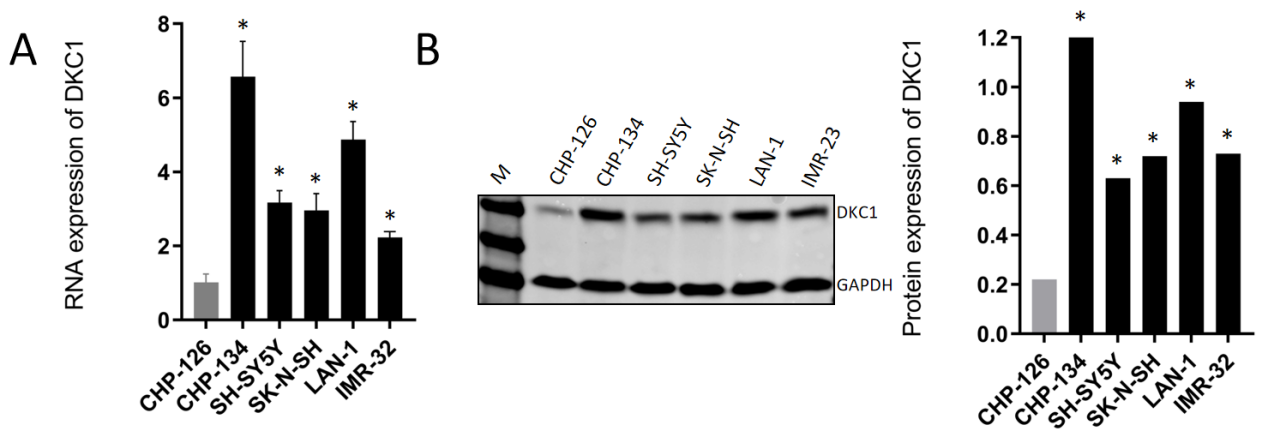


Figure S2 High expression of DKC1 in human neuroblastoma cell lines. A) mRNA expression level of DKC1 in different cell lines; B) Protein levels of DKC1 in different cell lines
